# Serum LH levels before progesterone administration significantly affect pregnancy outcomes in hormone replacement therapy-frozen-thawed embryo transfer cycles

**DOI:** 10.1186/s13048-025-01743-x

**Published:** 2025-07-19

**Authors:** Xu Han, Chang Liu, Jie Wang, Ye Zheng, Huidan Wang, Mei Sun, Xiufang Li

**Affiliations:** 1 State Key Laboratory of Reproductive Medicine and Offspring Health, Center for Reproductive Medicine, Institute of Women, Children and Reproductive Health, Shandong University, 157 Jingliu Road, Jinan, 250012 Shandong Province China; 2 National Research Center for Assisted Reproductive Technology and Reproductive Genetics, Shandong University, Jinan, 250012 Shandong China; 3 Key Laboratory of Reproductive Endocrinology (Shandong University), Ministry of Education, Jinan, 250012 Shandong China; 4 Shandong Technology Innovation Center for Reproductive Health, Jinan, 250012 Shandong China; 5 Shandong Provincial Clinical Research Center for Reproductive Health, Jinan, 250012 Shandong China; 6 Shandong Key Laboratory of Reproductive Research and Birth Defect Prevention, Jinan, 250012 Shandong China; 7 Research Unit of Gametogenesis and Health of ART-Offspring, Chinese Academy of Medical Sciences (No.2021RU001), Jinan, 250012 Shandong China; 8 Shandong Provincial Hospital Affiliated to Shandong First Medical University, Jinan, Shandong China; 9 Shandong Provincial Hospital, Shandong University, Jinan, Shandong China

**Keywords:** Hormone replacement therapy, Frozen-thawed embryo transfer, Live birth rate, Pregnancy outcome, Luteinizing hormone

## Abstract

**Background:**

In hormone replacement therapy-frozen-thawed embryo transfer (HRT-FET) cycles, endogenous LH levels may still rise, and the relationship between this and pregnancy outcomes is unclear. The purpose of this study was to investigate the correlation between the serum LH levels before progesterone administration in HRT-FET cycles and the live birth rate (LBR).

**Methods:**

A total of 13 720 HRT-FET cycles were divided into four groups based on serum LH levels according to the quartiles. Meanwhile, subgroup analyses were performed based on the use of pituitary down-regulation to evaluate the independent effects of serum LH levels on pregnancy outcomes. We used multivariate logistic regression analysis to adjust for potential confounding factors.

**Results:**

In the overall, the 51-75th percentile group showed significant differences in LBR and miscarriage rate compared to the reference group (*P* = 0.010; *P* = 0.004), and the > 75th percentile group showed significant difference in biochemical pregnancy rate compared to the reference group (*P* = 0.022). In the non-pituitary down-regulation group, the 51-75th percentile group and the reference group exhibited significant differences in LBR and miscarriage rate (*P* = 0.004), and the 26–50th percentile group showed significant difference in miscarriage rate compared to the reference group (*P* = 0.026). In the pituitary down-regulation group, the > 75th percentile group showed significant difference in biochemical pregnancy rate compared to the reference group (*P* = 0.045).

**Conclusion:**

In HRT-FET cycles, low serum LH levels prior to progesterone administration may be associated with poor pregnancy outcomes. For patients presenting with low LH levels, we may recommend deferring the FET cycle to reduce poor pregnancy outcomes.

**Supplementary Information:**

The online version contains supplementary material available at 10.1186/s13048-025-01743-x.

## Background

In assisted reproductive technology, it is now more common to culture all embryos to the expanded blastocyst stage and then freeze them [1]. And the proportion of frozen-thawed embryo transfer (FET) in in vitro fertilization (IVF) cycles is increasing [2], thereby reducing the incidence of complications such as ovarian hyperstimulation. Endometrium preparation protocols for FET are primarily categorized into two groups: those involving ovulation and those without ovulation. However, a consensus regarding the optimal endometrium preparation protocol for achieving the most favorable pregnancy outcome has yet to be established [3–7].

The hormone replacement therapy (HRT)-FET protocol primarily stimulates endometrial growth through exogenous estrogen supplementation, and then administer exogenous progesterone when the endometrial thickness reaches an adequate level [8]. The timing of FET is determined by progesterone administration [9]. In contrast, natural cycle FET necessitates ovulation monitoring to determine the optimal timing for FET; however, unobserved ovulations can lead to cycle cancellation [10, 11]. Therefore, compared to natural cycle FET, HRT-FET may have a lower rate of cycle cancellation and fewer patient visits, potentially resulting in reduced healthcare costs.

Luteinizing hormone (LH) is a hormone released by the pituitary gland in response to the stimulation of gonadotropin-releasing hormone (GnRH), which plays an important role in ovulation, preparing the uterus for implantation of a fertilized egg, and producing estrogen and progesterone in the ovaries [12]. The function of LH in the ovary is well-established through its binding to the LH/chorionic gonadotropin receptor (LHCGR) [13]. During the luteal phase, LH is capable of enhancing luteal function and stimulating the granulosa cell’s secretion of progesterone [12]. Previous studies have identified the LHCGR expression in the uterus, suggesting that LH may influence endometrial receptivity and placenta formation by binding to LHCGR in this organ [14–16]. While the significant role of LH in natural cycle FET has been extensively investigated [17], its involvement in HRT-FET remains relatively understudied. In HRT-FET cycles, although dominant follicle development does not occur, exogenous estrogen supplementation promotes endometrial proliferation accompanied by an increase in the LH level. However, the mechanism underlying this increase remains unclear. Additionally, there is limited consensus regarding how elevated LH levels impact pregnancy outcomes in HRT-FET cycles. Some studies have suggested that low LH levels are associated with the poor pregnancy outcomes, while others have indicated no significant effect [18–21].

Therefore, the objective of this study was to further validate the predictive value of serum LH levels for pregnancy outcomes in HRT-FET cycles by employing a larger sample size and conducting subgroup analyses based on pituitary down-regulation status.

## Materials and methods

### Study design and participants

**Fig. 1:**
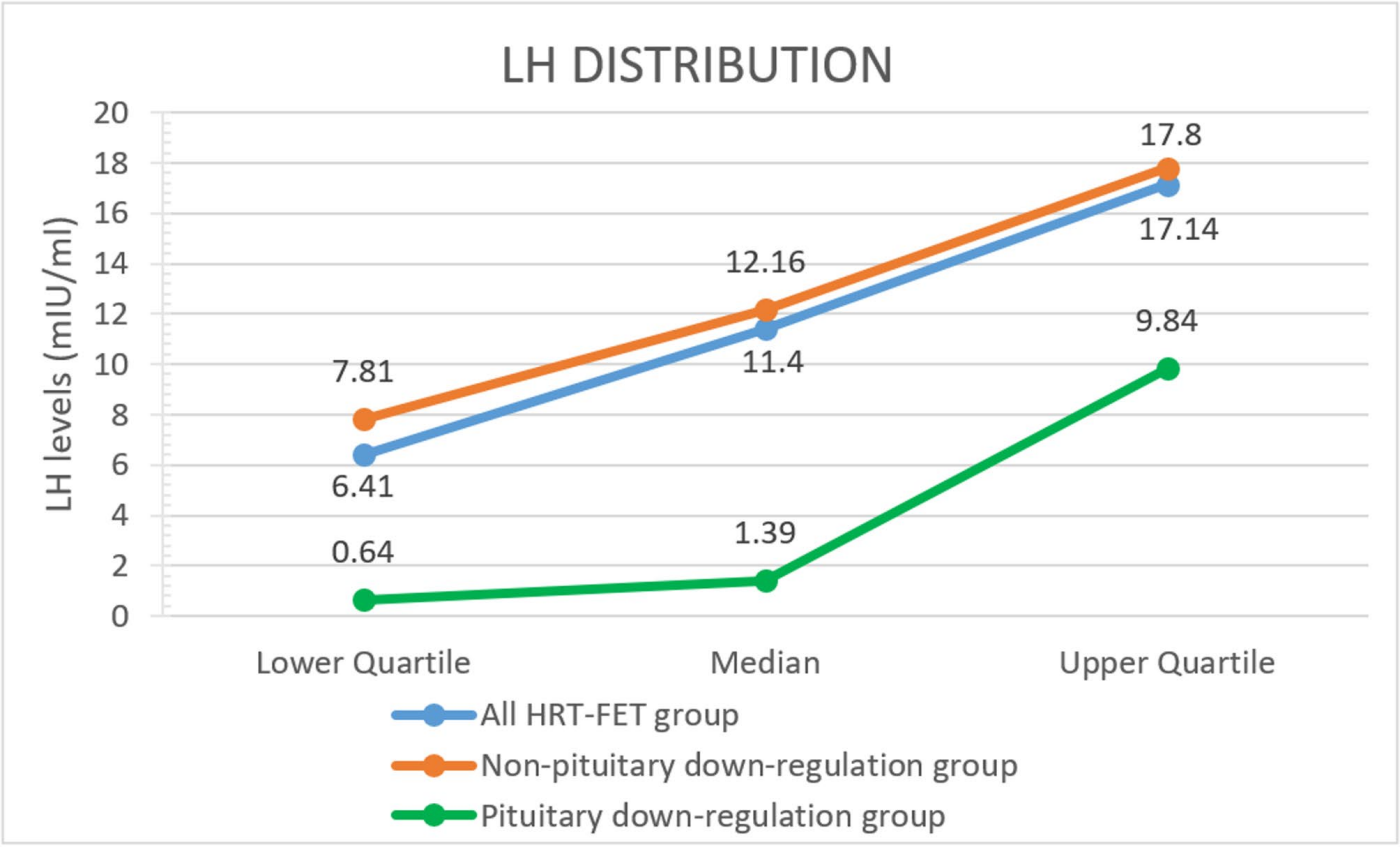
LH Distribution Trend, LH: Luteinizing Hormone; HRT-FET, hormone replacement therapy-frozen-thawed embryo transfer

Retrospective cohort study design was employed in this investigation, with the Center for Reproductive Medicine, Shandong University being selected as the research site. The inclusion criteria consisted of patients who underwent FET between January 2015 and January 2022, meeting the following inclusion criteria: (i) undergoing IVF or intracytoplasmic sperm injection (ICSI) cycles; (ii) performing frozen-thawed blastocyst transfer; (iii) employing HRT cycles for endometrial preparation. Exclusion criteria were as followed: (i) preimplantation genetic testing (PGT) cycles; (ii) with a history of uterine anomalies such as intrauterine adhesions and uterine malformation et al.; (iii) hydrosalpinx. According to the quartiles of serum LH levels prior to progesterone administration, they were categorized into four groups (Fig. 1): ≤25th percentile (LH ≤ 6.41 mIU/ml), 26-50th percentile (6.41 < LH ≤ 11.40 mIU/ml), 51-75th percentile (11.40 < LH ≤ 17.14 mIU/ml), and > 75th percentile (LH > 17.14 mIU/ml). In the non-pituitary down-regulation group, the distribution across these groups was as follows: ≤25th percentile (LH ≤ 7.81 mIU/ml), 26-50th percentile (7.81 < LH ≤ 12.16 mIU/ml), 51-75th percentile (12.16 < LH ≤ 17.80 mIU/ml), and > 75th percentile (LH > 17.80 mIU/ml). In the pituitary down-regulation group, the distribution across these groups was as follows: ≤25th percentile (LH ≤ 0.64 mIU/ml), 26-50th percentile (0.64 < LH ≤ 1.39 mIU/ml), 51-75th percentile (1.39 < LH ≤ 9.84 mIU/ml), and > 75th percentile (LH > 9.84 mIU/ml). The reference group was defined as those with LH levels ≤ the 25th percentile.

### Endometrial preparation

For the non-pituitary down-regulation group, all patients underwent transvaginal ultrasound examination within the first 3 days of menstruation, followed by oral administration of estradiol valerate (Progynova, Bayer-Schering Pharma AG, Germany). For the pituitary down-regulation group, patients received a 3.75 mg GnRH agonist (GnRH-a) injection within the first 3 days of menstruation, followed by transvaginal ultrasound examination approximately 4–5 weeks later, after which oral estradiol valerate was initiated. The initial dose of estradiol for both protocols was 4 mg daily for 5 days, then increased to 6 mg for the next 5 days. Subsequently, a follow-up visit was scheduled for transvaginal ultrasound and blood tests to assess estrogen, progesterone, and serum LH levels. Further management was determined based on endometrial thickness and serum estradiol levels, with options to either continue estradiol valerate (up to a maximum dose of 8 mg daily) or initiate progesterone supplementation. Luteal phase support with oral dydrogesterone 40 mg daily (Abbott, Netherlands) and vaginal progesterone capsules 200 mg daily (Laboratoires Besins International, France) were added once the endometrial thickness reached 6.5 mm or more, and FET was performed 5 days later. If pregnancy occurred, estrogen and progesterone needed to be maintained until the placenta was fully developed to compensate for the lack of the corpus luteum.

### Embryo morphology evaluation, thawing and transfer

Blastocyst morphology was assessed using the Gardner grading system [22], categorized into six stages based on blastocoel development (Supplemental Table 1) [23]. Blastocysts scoring stage 4 (4BC) or higher were defined as high-quality blastocysts. Typically, 1 to 2 embryos were transferred per cycle, with thawing usually performed in the morning and embryo transferred in the afternoon.

### Outcome measures

The primary outcome of this study was the live birth rate (LBR), while the secondary outcomes included biochemical pregnancy rate, clinical pregnancies rate, miscarriage rate, and ectopic pregnancies rate. Subgroup analysis was performed based on pituitary down-regulation status, as GnRH-a pretreatment may significantly alter LH secretion patterns. Biochemical pregnancy was defined as a serum β-human chorionic gonadotropin (β-hCG) levels ≥ 25 IU/L at 12 days after embryo transfer. Clinical pregnancy was defined as the presence of a gestational sac visible in the uterine cavity through transvaginal ultrasound at about 35 days after embryo transfer. Miscarriage was defined as the occurrence of spontaneous or therapeutic abortions before 28 weeks of gestation. Ectopic pregnancy was defined as a gestation implanted outside the uterine cavity, including tubal, ovarian, cervical, cornual, or abdominal locations, as confirmed by either transvaginal ultrasound or surgical intervention. Live birth was defined as the delivery of the viable neonate who was 28 weeks of gestation or older, in accordance with the standard established by the National Health Commission of the People’s Republic of China.

### Statistical analysis

Statistical analyses were conducted using SPSS 26.0 software. The Kolmogorov-Smirnov test was employed to assess the normal distribution of continuous variables. For variables violating the normality assumption, the Bootstrap resampling method was employed to evaluate result stability. For normally distributed continuous variables, data were summarized as mean ± standard deviation and analyzed by one-way analysis of variance, whereas non-normally distributed variables were reported as median (interquartile ranges) and compared via the Kruskal-Wallis test. Categorical variables were described as percentages and compared using the chi-square test. Logistic regression analysis was utilized to identify potential confounding variables that might independently influence pregnancy outcomes. Confounding factors underwent univariate analysis for testing before being adjusted in the multivariate regression model. A *P*-value < 0.05 was considered statistically significant.

## Results

**Study population and basal characteristics**.

**Fig. 2:**
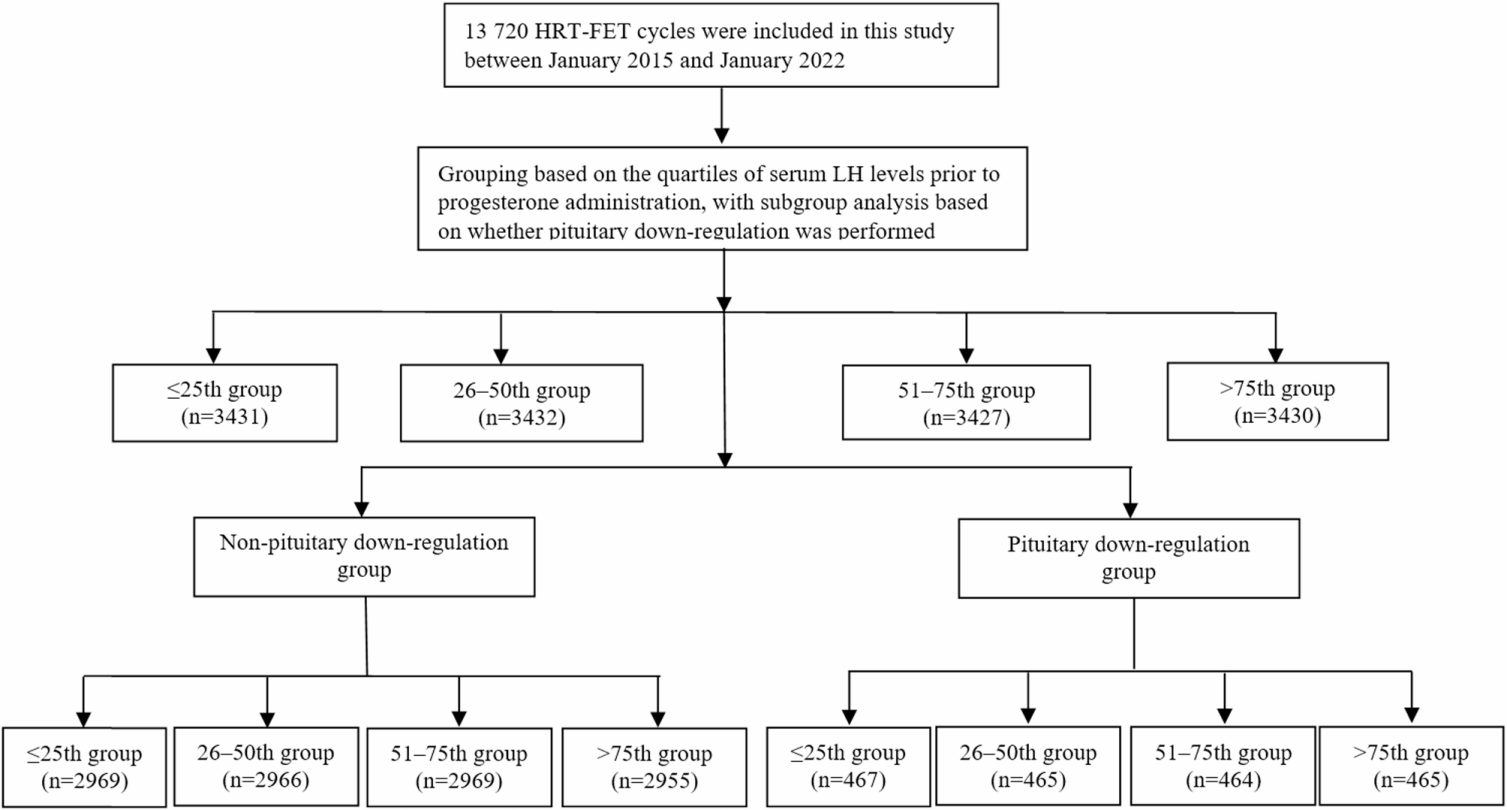
Flowchart and data processing. HRT-FET, hormone replacement therapy-frozen-thawed embryo transfer

A total of 13 720 HRT-FET cycles were included in this study, encompassing both non-pituitary down-regulation and pituitary down-regulation groups (Fig. 2). According to the quartiles of serum LH levels prior to progesterone administration, the FET cycles were categorized into four groups: ≤25th percentile, > 25th to ≤ 50th percentile, > 50th to ≤ 75th percentile, and > 75th percentile.

The baseline characteristics of the patients are presented in Table 1. There were significant differences between the two groups in terms of age, basal follicle-stimulating hormone, basal LH, basal estradiol, anti-Müllerian hormone, body mass index (BMI), types of infertility, causes of infertility, numbers of retrieved oocytes, fertilization mode, estradiol, LH and progesterone levels before progesterone administration, number of embryos transferred cycles and development days of blastocysts. However, no significant differences were found between the two groups regarding endometrial thickness before progesterone administration and the number of embryos transferred per cycle.

**Table 1:** Baseline demographics and cycle characteristics

	Serum LH levels prior to progesterone administration stratified by quartiles				
	≤ 25th (*n* = 3431)	26–50th (*n* = 3432)	51–75th (*n* = 3427)	> 75th (*n* = 3430)	*P*-value
Age (years)	31.33 ± 4.59	30.88 ± 4.60	30.71 ± 4.52	30.48 ± 4.57	< 0.001
Basal hormone					
FSH (mIU/ml)	6.30 ± 2.13	6.10 ± 1.86	6.20 ± 1.93	6.44 ± 1.95	< 0.001
LH (mIU/ml)	5.93 ± 3.83	6.58 ± 4.28	7.01 ± 4.58	7.34 ± 4.74	< 0.001
Estradiol (pg/ml)	36.35 ± 15.72	35.39 ± 14.41	36.50 ± 15.30	37.01 ± 15.02	< 0.001
AMH (ng/ml)	4.12 (2.07, 7.39)	5.13 (2.71, 9.02)	5.27 (2.87, 8.94)	5.04 (2.68, 8.81)	< 0.001
Types of infertility					0.043
Primary	50.2% (1724/3431)	50.3% (1726/3432)	52.9% (1812/3427)	52.5% (1801/3430)	
Secondary	49.8% (1707/3431)	49.7% (1706/3432)	47.1% (1615/3427)	47.5% (1629/3430)	
BMI (kg/m^2^)	24.49 ± 3.61	24.67 ± 3.67	24.35 ± 3.59	23.63 ± 3.42	< 0.001
Causes of infertility					< 0.001
Ovulatory Factor	26.1% (894/3431)	36.7% (1258/3432)	34.6% (1187/3427)	32.2% (1103/3430)	
Tubal factor	55.4% (1900/3431)	48.4% (1600/3432)	47.4% (1624/3427)	49.7% (1704/3430)	
Endometriosis	1.6% (56/3431)	0.5% (17/3432)	0.7% (24/3427)	0.5% (16/3430)	
Male factor	5.6% (191/3431)	6.2% (213/3432)	7.5% (256/3427)	6.9% (236/3430)	
Other	11.4% (390/3431)	8.3% (284/3432)	9.8% (336/3427)	10.8% (371/3430)	
Numbers of oocytes retrieved	13.49 ± 7.13	14.76 ± 7.35	14.99 ± 7.42	14.46 ± 7.61	< 0.001
Fertilization mode					0.014
IVF	60.8% (2086/3431)	59.5% (2041/3432)	59.0% (2022/3427)	57.0% (1955/3430)	
ICSI	39.2% (1345/3431)	40.5% (1391/3432)	41.0% (1405/3427)	43.0% (1475/3430)	
Prior to progesterone administration					
Endometrial thickness (cm)	0.95 ± 0.15	0.94 ± 0.14	0.94 ± 0.14	0.94 ± 0.14	0.149
Estradiol (pg/ml)	179.65 (140.00, 240.00)	179.40 (139.60, 234.60)	175.00 (135.00, 226.80)	175.00 (134.90, 230.50)	0.028
Progesterone (ng/ml)	0.15 (0.08, 0.27)	0.19 (0.10, 0.35)	0.19 (0.11, 0.35)	0.22 (0.12, 0.39)	< 0.001
LH (mIU/ml)	1.12 (0.38, 4.34)	9.00 (7.87, 10.20)	13.84 (12.55, 15.39)	22.45 (19.53, 23.90)	< 0.001
Number of embryos transferred cycles	1.47 ± 0.78	1.34 ± 0.71	1.30 ± 0.70	1.29 ± 0.66	< 0.001
Number of embryos transferred per cycle	1.06 ± 0.23	1.06 ± 0.24	1.06 ± 0.23	1.06 ± 0.24	0.537
Development days of blastocysts					0.029
Day 5	72.0% (2470/3431)	73.6% (2527/3432)	74.1% (2538/3427)	75.1% (2577/3430)	
Day 6	28.0% (961/3431)	26.4% (905/3432)	25.9% (889/3427)	24.9% (853/3430)	

FSH, follicle-stimulating hormone; LH, luteinizing hormone; AMH, anti-Müllerian hormone; BMI, body mass index; IVF, in vitro fertilization; ICSI, intracytoplasmic sperm injection

### Pregnancy outcomes

The overall and subgroup univariate analyses of pregnancy outcomes among the different groups are presented in Table 2. In the overall analysis, there were significant differences in LBR, biochemical pregnancy rate, clinical pregnancy rate, and miscarriage rate between the groups (*P* < 0.001, *P* = 0.022, *P* = 0.022, *P* < 0.001), while no significant difference was found in ectopic pregnancy rate. In the subgroup analysis, significant differences were observed in LBR and miscarriage rate among the groups in the non-pituitary down-regulation group (*P* = 0.005, *P* < 0.001), whereas no significant differences were observed in other pregnancy outcomes. In the pituitary down-regulation group, no significant differences in pregnancy outcomes were detected among the groups.

**Table 2:** Comparison of pregnancy outcomes between groups: univariate analysis both overall and subgroup

	Serum LH levels prior to progesterone administration stratified by quartiles				
	≤ 25th	26–50th	51–75th	> 75th	*P*-value
Overall					
Live birth rate	45.2% (1552/3431)	47.6% (1635/3432)	49.8% (1705/3427)	49.7% (1703/3430)	< 0.001
Biochemical pregnancy rate	65.2% (2238/3431)	66.4% (2280/3432)	67.1% (2299/3427)	68.7% (2356/3430)	0.022
Clinical pregnancy rate	56.3% (1933/3431)	57.8% (1983/3432)	58.9% (2017/3427)	59.9% (2053/3430)	0.022
Miscarriage rate	16.8% (324/1933)	12.8% (254/1983)	11.4% (230/2017)	12.8% (262/2053)	< 0.001
Ectopic pregnancy rate	0.7% (13/1933)	0.3% (6/1983)	0.7% (14/2017)	0.8% (16/2053)	0.222
Non-pituitary down-regulation group					
Live birth rate	46.4% (1379/2969)	47.9% (1422/2966)	50.6% (1502/2969)	49.9% (1476/2955)	0.005
Biochemical pregnancy rate	66.9% (1986/2969)	66.5% (1973/2966)	67.7% (2009/2969)	68.4% (2022/2955)	0.407
Clinical pregnancy rate	57.8% (1717/2969)	57.7% (1712/2966)	60.1% (1785/2969)	59.5% (1758/2955)	0.151
Miscarriage rate	16.0% (274/1717)	12.2% (209/1712)	11.5% (205/1785)	11.7% (206/1758)	< 0.001
Ectopic pregnancy rate	0.7% (12/1717)	0.2% (3/1712)	0.7% (13/1785)	0.6% (10/1758)	0.103
Pituitary down-regulation group					
Live birth rate	41.8% (195/467)	45.6% (212/465)	43.8% (203/464)	44.3% (206/465)	0.695
Biochemical pregnancy rate	60.6% (283/467)	63.4% (295/465)	63.8% (296/464)	66.5% (309/465)	0.326
Clinical pregnancy rate	51.2% (239/467)	56.3% (262/465)	54.3% (252/464)	56.1% (261/465)	0.359
Miscarriage rate	16.7% (40/239)	16.8% (44/262)	17.1% (43/252)	18.8% (49/261)	0.918
Ectopic pregnancy rate	0.8% (2/239)	0.8% (2/262)	0.4% (1/252)	2.3% (6/261)	0.166

LH, luteinizing hormone

**Fig. 3:**
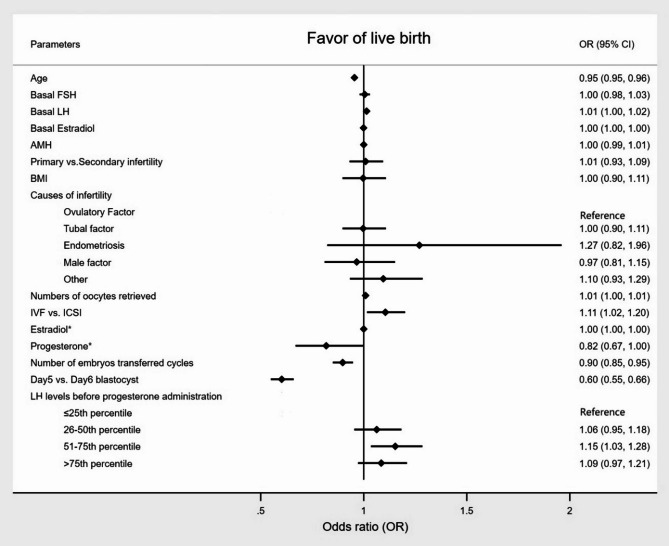
Multivariable logistic regression analysis for live birth. FSH, follicle-stimulating hormone; LH, luteinizing hormone; AMH, anti-Müllerian hormone; BMI, body mass index; IVF, in vitro fertilization; ICSI, intracytoplasmic sperm injection. *The day before progesterone administration

The overall and subgroup multivariable logistic regression analyses of pregnancy outcomes among the different groups are presented in Table 3. In the overall, the 51-75th percentile group showed significant differences in LBR (Fig. 3) and miscarriage rate compared to the reference group (adjusted odds ratio [OR] = 1.153, 95% confidence interval [CI] 1.035–1.285, *P* = 0.010; adjusted OR = 0.734, 95% CI 0.595–0.905, *P* = 0.004), and the > 75th percentile group showed significant difference in biochemical pregnancy rate compared to the reference group (adjusted OR = 1.146, 95% CI 1.020–1.288, *P* = 0.022). While no significant differences were found in other pregnancy outcomes. In the subgroup analysis, the 51-75th percentile group and the reference group exhibited significant differences in LBR and miscarriage rate (adjusted OR = 1.166, 95% CI 1.039–1.308, *P* = 0.009; adjusted OR = 0.719, 95% CI 0.575–0.899, *P* = 0.004), and the 26–50th percentile group showed significant difference in miscarriage rate compared to the reference group (adjusted OR = 0.779, 95% CI 0.625–0.970, *P* = 0.026) in the non-pituitary down-regulation group; no other significant differences were observed for other pregnancy outcomes among the different groups. In the pituitary down-regulation group, the > 75th percentile group showed significant difference in biochemical pregnancy rate compared to the reference group (adjusted OR = 1.378, 95% CI 1.008–1.884, *P* = 0.045), whereas no significant differences were observed for other pregnancy outcomes among the different groups.

**Table 3:** Comparison of pregnancy outcomes between groups: multivariable logistic regression analysis both overall and subgroup

	Serum LH levels prior to progesterone administration stratified by quartiles									
	≤ 25th	26–50th			51–75th			> 75th		
		OR	95%CI	*P*-value	OR	95%CI	*P*-value	OR	95%CI	*P*-value
Overall										
Live birth rate	Ref	1.063	0.954–1.183	0.270	1.153	1.035–1.285	0.010	1.085	0.972–1.121	0.145
Biochemical pregnancy rate	Ref	1.041	0.929–1.166	0.489	1.074	0.958–1.203	0.223	1.146	1.020–1.288	0.022
Clinical pregnancy rate	Ref	1.042	0.934–1.161	0.460	1.088	0.976–1.214	0.129	1.100	0.985–1.229	0.092
Miscarriage rate	Ref	0.835	0.682–1.022	0.080	0.734	0.595–0.905	0.004	0.953	0.777–1.169	0.647
Ectopic pregnancy rate	Ref	0.674	0.218–2.079	0.492	1.492	0.591–3.769	0.398	1.712	0.691–4.240	0.245
Non-pituitary down-regulation group										
Live birth rate	Ref	1.073	0.957–1.203	0.230	1.166	1.039–1.308	0.009	1.072	0.953–1.205	0.246
Biochemical pregnancy rate	Ref	0.990	0.877–1.118	0.877	1.032	0.913–1.167	0.615	1.075	0.948–1.218	0.259
Clinical pregnancy rate	Ref	1.015	0.904–1.140	0.801	1.099	0.977–1.235	0.115	1.045	0.927–1.176	0.472
Miscarriage rate	Ref	0.779	0.625–0.970	0.026	0.719	0.575–0.899	0.004	0.889	0.711–1.111	0.300
Ectopic pregnancy rate	Ref	0.454	0.116–1.768	0.255	1.343	0.493–3.664	0.564	1.347	0.486–3.735	0.567
Pituitary down-regulation group										
Live birth rate	Ref	1.118	0.828–1.510	0.466	1.014	0.751–1.370	0.926	1.139	0.843–1.539	0.398
Biochemical pregnancy rate	Ref	1.056	0.776–1.437	0.728	1.152	0.845–1.559	0.370	1.378	1.008–1.884	0.045
Clinical pregnancy rate	Ref	1.177	0.870–1.590	0.290	1.086	0.804–1.466	0.591	1.260	0.931–1.704	0.134
Miscarriage rate	Ref	0.994	0.575–1.717	0.981	1.155	0.672–1.984	0.602	1.181	0.691–2.020	0.543
Ectopic pregnancy rate	Ref	0.369	0.030–4.480	0.434	0.391	0.03–4.763	0.431	2.054	0.345–12.241	0.429

LH, luteinizing hormone

## Discussion

In HRT-FET cycles, serum LH levels may be associated with pregnancy outcomes, but the conclusions of several studies remain inconsistent. The results of this study indicate that low serum LH levels reduce the LBR and increase the miscarriage rate in HRT-FET cycles. Additionally, this study conducted subgroup analyses based on the use of pituitary down-regulation. The findings in non-pituitary down-regulation group were consistent with the aforementioned results, whereas in pituitary down-regulation group, serum LH levels showed no association with LBR.

LH plays a crucial role in pregnancy and has been closely associated with pregnancy outcomes in IVF ovarian stimulation cycles [12, 24–26]. However, the impact of LH on pregnancy outcomes in HRT-FET cycles remains controversial. A retrospective cohort study [19] involving 3,469 HRT-FET cycles analyzed the correlation between LH levels on the day before progesterone administration and pregnancy outcomes. The study found that low LH levels were associated with a reduced LBR, and subgroup analysis based on BMI revealed that this correlation was significant only in women with a BMI of less than 25 kg/m². Similarly, Ismail Guler et al. [18] reported an association between low LH levels and decreased LBRs, suggesting that delaying estrogen initiation might influence LH levels and improve pregnancy outcomes. However, some studies have yielded findings that contradict these conclusions. A study [20] involving the first HRT-FET cycle of 513 patients found no correlation between LH levels and clinical pregnancy rate. It is important to note, however, that this study focused solely on clinical pregnancy rate and did not evaluate live birth or miscarriage rates. Another study [21] divided participants into two groups based on whether LH levels increased by onefold before estrogen administration and progesterone administration, and the results showed that the LH elevation during the HRT-FET cycles did not affect pregnancy outcomes. This study primarily examined the impact of LH level changes on pregnancy outcomes and required the measurement of basal LH levels before estrogen administration for comparison. Nevertheless, due to inherent limitations such as small sample sizes, the reliability of these findings may be limited. In contrast, our study included the largest sample size to date to further evaluate the correlation between serum LH levels and pregnancy outcomes. The results demonstrated that low serum LH levels were associated with an increased risk of miscarriage and a decreased LBR, aligning with some previous research findings.

Existing research findings on whether pituitary down-regulation in HRT-FET cycles improves pregnancy outcomes remained inconsistent [4, 27, 28]. Based on previous study results, it was hypothesized that the use of pituitary down-regulation in HRT-FET cycles might adversely affect pregnancy outcomes due to low LH levels. However, there was currently a lack of research investigating the potential impact of LH levels in pituitary down-regulation HRT-FET cycles on pregnancy outcomes. Through subgroup analysis, this study found no association between serum LH levels and LBR in pituitary down-regulation HRT-FET group. The mechanisms and dynamic patterns of LH elevation in HRT-FET cycles remain poorly characterized. GnRH-a-mediated suppression of endogenous LH secretion may mask the physiological effects of LH, while the uniformly suppressed LH levels across the groups could account for the absence of LBR differences in the pituitary down-regulation subgroup. Moreover, due to the limited sample size in the pituitary down-regulation subgroup, particularly for outcomes such as ectopic pregnancy rate, the findings may be underpowered. Therefore, these results should be interpreted with caution, and further validation through larger-scale studies or prospective designs is warranted.

The administration of GnRH-a analogues during ovarian stimulation cycles may result in excessive suppression of pituitary function, leading to insufficient LH levels. Consequently, there has been an increasing number of studies investigating whether exogenous LH supplementation could improve pregnancy outcomes in IVF cycles undergoing controlled ovarian stimulation. Most research findings suggested that adding exogenous LH in ovarian stimulation cycles may be helpful in improving pregnancy outcomes for poor responders and advanced age women [29–31]. Although the mechanism through which LH affects pregnancy outcomes in HRT-FET cycles is not fully understood, it may be achieved by facilitating the transformation of the endometrium during the luteal phase, enhancing endometrial receptivity, and promoting embryo implantation potential [32–34]. LH can induce intracellular cyclic adenosine monophosphate accumulation and activate key steroidogenic enzymes such as aromatase and P450 cholesterol side-chain cleavage enzyme, which are essential for estrogen and progesterone synthesis, thereby indirectly influencing endometrial thickness and receptivity [35]. A study demonstrated that low LH levels downregulated mitochondrial-nicotinamide adenine dinucleotide metabolism, glycolytic metabolism, immune regulation, and autophagic function, thereby impairing endometrial receptivity [36]. Consequently, this study demonstrated a significant association between low serum LH levels and adverse pregnancy outcomes, potentially mediated by impaired endometrial receptivity resulting from LH deficiency. For patients presenting with reduced LH levels, we may recommend deferring the FET cycle to reduce the likelihood of poor pregnancy outcomes. However, given the inherent limitations of this retrospective analysis, clinical decisions should be made after comprehensive evaluation of each patient’s individual circumstances to provide personalized recommendations. Simultaneously, the potential benefits of exogenous LH supplementation in improving pregnancy outcomes for patients with low LH levels during HRT-FET cycles deserve further investigation.

The primary strength of this study lies in its single-center design, which incorporates the largest sample size reported to date in this field. Unlike previous studies that measured LH levels on a predefined fixed day, the LH levels included in this study were within a window of 5 to 8 days prior to FET. This design was primarily driven by the fact that LH measurements were routinely conducted only during the initial follow-up visit after the initiation of estrogen replacement therapy, thereby reducing both the frequency of patient visits and associated healthcare costs. However, this also represents a limitation of the present study. A single measurement may introduce bias, as we were unable to determine the dynamic changes in LH levels during the HRT-FET cycle, which may exhibit a biphasic pattern characterized by an initial rise followed by a subsequent decline. This could potentially compromise the reliability of the conclusions. Therefore, future studies may need to focus more on the variations in LH levels, and further investigations into the underlying mechanisms are also warranted. Furthermore, due to the inherent limitations of this retrospective study design in establishing a causal relationship between LH levels and pregnancy outcomes, the absence of statistically significant differences in clinical pregnancy rates and ectopic pregnancy rates does not preclude the possibility of underlying biological associations. Although these clinically meaningful findings did not reach the threshold of statistical significance, they warrant further validation through prospective studies to confirm the results and elucidate potential mechanistic relationships.

## Conclusion

This study demonstrated that low LH levels were significantly associated with a decreased LBR and an increased risk of miscarriage. Thus, serum LH levels could serve as a valuable predictor of pregnancy outcomes in HRT-FET cycles. For patients presenting with low LH levels, we may recommend deferring the FET cycle to reduce poor pregnancy outcomes; however, as this was a retrospective study, clinical decisions should also incorporate the patient’s individual history and characteristics. The precise mechanism by which LH fluctuations influence pregnancy outcomes in HRT-FET cycles remained unclear. In future research, further mechanistic studies and randomized controlled trials are necessary for confirmation.

## Electronic supplementary material

Below is the link to the electronic supplementary material.

Supplementary Material 1

## Data Availability

The data underlying this article will be shared on reasonable request to the corresponding author.
