# Supplementary material for: Serum LH levels before progesterone administration significantly affect pregnancy outcomes in hormone replacement therapy-frozen-thawed embryo transfer cycles
Source: J Ovarian Res. 2025 Jul 19;18:158. doi: 10.1186/s13048-025-01743-x (PMC12275408; doi:10.1186/s13048-025-01743-x)
Supplement: Supplementary file 1 — Supplementary Material 1 [file 13048_2025_1743_MOESM1_ESM.docx]

Supplemental Table 1. Blastocyst morphology evaluation

|  | Grade | Description |
| --- | --- | --- |
| Developmental Stage | Stage 1 | Early cavitary blastocyst with blastocyst cavity less than 1/2 of the embryo volume |
|  | Stage 2 | blastocyst cavity coelom greater than or equal to 1/2 of the volume |
|  | Stage 3 | fully expanded blastocyst, blastocyst cavity occupies the embryo |
|  | Stage 4 | expanded blastocyst, blastocyst cavity volume greater than an early embryo, zona pellucida thinning |
|  | Stage 5 | blastocyst being hatched, trophoblast begins to break through the zona pellucida |
|  | Stage 6 | hatched blastocyst, the blastocyst is completely hatched from the zona pellucida |
| Inner cell mass (ICM) | A | the inner cell mass is tight and the number of cells is large |
|  | B | the inner cell mass is loose and the number is small |
|  | C | the number of the inner cell mass is very small |
| trophectoderm (TE) | A | the number of cells is large and a tightly arranged cell layer is formed |
|  | B | the number of cells is small and the arrangement is loose |
|  | C | the trophectoderm is composed of sparse cells |
